# Sex differences in intracranial plaque burden in patients with type 2 diabetes mellitus with acute ischemic cerebrovascular disease: a pilot study based on high-resolution MRI

**DOI:** 10.3389/fendo.2024.1417240

**Published:** 2025-01-24

**Authors:** Xuejiao Yan, Ling Li, Jie Gao, Lihui Wang, Kai Ai, Xiaoyan Lei, Min Tang, Xiaoling Zhang, Dongsheng Zhang

**Affiliations:** ^1^ Department of Magnetic Resonance Imaging (MRI), Shaanxi Provincial People’s Hospital, Xi’an, Shaanxi, China; ^2^ Department of Radiology, Xi‘an International Medical Center Hospital, Xi’an, Shaanxi, China; ^3^ Department of Clinical Science, Philips Healthcare, Xi’an, China

**Keywords:** diabetes mellitus, sex difference, ischemic stroke, cardiovascular magnetic resonance imaging, intracranial atherosclerosis

## Abstract

**Background:**

Atherosclerosis (AS) is the main cause of macrovascular disease. Previous studies have found sex differences in the prevalence of type 2 diabetes mellitus (T2DM) and its associated macrovascular disease outcomes. However, the relationship between sex differences, T2DM, and AS is not fully understood. This study attempts to explore possible associations between sex, treatment, and the burden of intracranial atherosclerosis (ICAS) in patients with T2DM who have experienced an acute ischemic cerebrovascular disease.

**Methods:**

We focused on patients with T2DM with acute ischemic stroke or transient ischemic attack due to intracranial atherosclerotic stenosis. ICAS was assessed by 3T cardiovascular magnetic resonance vascular wall imaging. Plaque counts of the total, proximal, and distal intracranial arteries were used to assess plaque burden. Patients with a history of T2DM and currently taking hypoglycemic drugs were defined as being treated. Poisson regression models or negative binomial regression models were used to analyze the interaction between sex and treatment in relation to plaque burden.

**Results:**

A total of 495 plaques were detected in 120 patients (75 male; mean age, 60.77 ± 11.01 years), including 311 proximal and 184 distal plaques. The intracranial culprit plaque was located proximal to the artery in both male (85.3%) and female (88.9%) patients. The adjusted total and proximal intracranial plaque burdens were 1.261 times (95% confidence interval [CI], 1.050–1.515, P=0.013) and 1.322 times (95%CI, 1.055–1.682, P=0.016) higher in male than in female patients. The risk ratio for proximal plaque burden in untreated male versus female patients was 0.966 (95%CI, 0.704–1.769). However, the proximal plaque risk ratio for treated male versus female patients was 1.530 (95%CI, 1.076–2.174). The interaction of sex and treatment significantly affected the proximal plaque burden.

**Conclusion:**

Male patients with T2DM and acute cerebrovascular disease have a significantly higher adjusted risk of total and proximal intracranial plaque burden compared to female patients. Female patients undergoing antidiabetic treatment have a significantly reduced risk of proximal plaque to males. Considering that culprit plaques tend to accumulate in the proximal arteries, understanding how to reduce the burden of proximal plaques may help reduce the risk of adverse cerebrovascular events.

## 1 Introduction

Type 2 diabetes mellitus (T2DM) affects nearly 9% of adults worldwide (1) and is one of the fastest growing diseases in China (2). This condition is closely associated with the occurrence, development, and severity of intracranial atherosclerosis (ICAS) (3), which is the main cause of ischemic cerebrovascular disease in the Chinese population (4). Hyperglycemic status, commonly observed in T2DM, promotes endothelial dysfunction in the early stages of atherosclerosis. ICAS may progress silently without any noticeable clinical symptoms, suggesting that early onset of T2DM could considerably influence the burden of ICAS and the risk of ischemic cerebrovascular disease in later life (5). Epidemiological studies in Asian populations have found that the prevalence of T2DM is higher in young to middle-aged males than in females (2), which may be related to insulin resistance being more pronounced in men from late adolescence to adulthood compared to females (6). However, with decreased sex-specific hormonal regulation and increased life expectancy, the prevalence of diabetes in women has gradually increased (7). Therefore, the effect of sex differences on the prevalence of T2DM on the burden of ICAS is not fully understood.

Effective management and control of T2DM, a major modifiable risk factor, may be an effective strategy to ultimately reduce the risk of ischemic cerebrovascular events. Studies on sex differences in treatment outcomes among patients with T2DM and cardiovascular disease, including myocardial infarction, stroke, and cardiovascular death, found that females with T2DM had a significantly lower risk of cardiovascular events compared to males (1). The relationship between the underlying atherosclerotic burden, a major cause of cardiovascular disease, and observed sex differences in cardiovascular risks of T2DM patients remains to be further investigated. Previous evaluations of atherosclerosis relied on luminal imaging techniques such as three-dimensional time-of-flight magnetic resonance angiography (MRA), enhanced MRA, or computed tomography angiography, and suggest ICAS when stenosis rates are ≥50% (4). Although the degree of arterial luminal stenosis may reflect the burden of atherosclerosis (8), extensive evidence suggests that the degree may be underestimated due to positive arterial remodeling effects (9) (thickening and compensatory outward expansion of the wall to maintain a normal luminal diameter) or overestimated due to partial volumetric effects caused by eddy currents (10). In recent years, advances in 3D cardiovascular magnetic resonance vascular wall imaging (CMR-VWI) have revolutionized the evaluation of ICAS. This technology provides detailed images of the vessel wall morphology and identifies plaques, overcoming the limitations of traditional vascular detection methods, and enables a more accurate evaluation of ICAS (11).

In this study, we used 3D CMR-VWI to investigate sex differences in the burden of intracranial atherosclerosis in patients with T2DM who have experienced acute cerebrovascular events. We hypothesized that males exhibit a higher burden of ICAS compared to females. In addition, we further analyzed the sex-specific effects of antidiabetic treatment on the burden of ICAS.

## 2 Materials and methods

### 2.1 Study population and design

The high-resolution MRI (HR-MRI) database, which focuses on high-resolution imaging of cerebral arteries in patients with acute ischemic stroke (AIS) or transient ischemic attack (TIA) from August 2019 to March 2024, was continuously reviewed. Patients underwent CMR-VWI after MRA detected intracranial artery stenosis or suspected stenosis. The detailed inclusion criteria were as follows: 1) AIS or TIA caused by ICAS, as determined by a neurologist; 2) Hospitalized patients with a glycated hemoglobin (HBA1c) level ≥6.5% or a fasting blood glucose level ≥7.0 mmol/L or history of T2DM; 3) Carotid artery or vertebral artery stenosis rate <50%; and 4) Undergoing CMR-VWI within two weeks of admission. The exclusion criteria were as follows: 1) Ischemic cerebral infarction caused by non-atherosclerotic vascular stenosis, such as moya moya disease, vasculitis, etc.; 2) Cardiogenic stroke; 3) Intracranial artery occlusion; 4) Receipt of intravascular intervention or thrombolytic therapy before CMR vascular wall imaging; 5) Patients undergoing sex hormone replacement therapy; 6) Poor image quality.

The study protocol was approved by Shaanxi Provincial People’s Hospital review board. All study participants provided signed informed consent.

### 2.2 Clinical and biochemical assessment

Clinical data and laboratory measurements including age, sex, height, weight, blood pressure, current smoking status, history of stroke, history of coronary heart disease, and medication were collected from the hospital’s electronic medical records. Laboratory tests performed 6 hours after admission on a fasting basis included measurements of total cholesterol, triglycerides, low-density lipoprotein cholesterol, high-density lipoprotein cholesterol (HDL-C), Apolipoprotein (Apo) A1, Apo B, uric acid (UA), homocysteine, HbA1c, and fasting blood glucose levels. Patients with a pre-admission history of type 2 diabetes mellitus and who were receiving antidiabetic drugs were defined as undergoing treatment.

### 2.3 MRI protocol

MRI scans were performed using the Philips 3.0T CMR scanner (Ingenia, Philips Medical System, The Netherlands) and a 16-channel head and neck coil. The CMR-VWI protocol included a three-dimensional (3D) time-of-flight (TOF) MRA and pre- and post-contrast 3D volume isotropic turbo spin-echo acquisition (VISTA). The 3D-TOF MRA was used to visualize vascular stenosis with the following parameters: repetition time (TR)/echo time (TE) = 20 ms/3.6 ms; field of view (FOV) = 180 × 180 mm^2^; matrix = 256 × 256; slice thickness = 5 mm. A 3D T_1_-weighted VISTA sequence of VW-MRI was obtained for plaque characteristic analysis with the following parameters: TR/TE = 700 ms/14 ms; FOV = 80 × 80 mm^2^; matrix = 256 × 256; slice thickness = 2 mm; layer spacing = 0.5 mm. The enhanced image was obtained using a repeated T_1_-weighted VISTA sequence after an intravenous injection of 0.1 mmol/kg contrast agent (Gadovist^®^, Bayer Schering Pharma AG, Berlin, Germany), followed by a delay of approximately 5 minutes. Diffusion-weighted imaging (DWI) after admission was used to identify acute cerebral infarction or TIA. The total duration for the complete sequence scan was approximately 20 minutes.

### 2.4 Image analysis

All MRI data were processed using semi-automatic software (tsimaging.net). The CMR-VWI images were evaluated by two neuroradiologists, X. Yan and L. Li, with six and five years of experience, respectively, who were blinded to clinical details. Atherosclerotic plaques on VW-MRI images showed eccentric thickening of the arterial wall, where the thinnest artery wall diameter was <50% of the thickest diameter. Each plaque was classified as either proximal (located in segments A1/M1/P1 of the anterior/middle/posterior cerebral artery and the origin of the basilar artery (BA) to the midpoint of the line from the superior cerebellar artery (SCA) to the anterior inferior cerebellar artery (AICA)) or distal (located in the anterior cerebral artery A2, posterior cerebral artery P2, middle cerebral artery M2-M3 segments and the midpoint of the line from the SCA to the AICA to the end of the BA). The sum of the proximal and distal plaque counts was considered the total plaque burden. In patients with stroke, the culprit plaque was defined as the only lesion in the same vascular area or the narrowest lesion when multiple plaques were present in the same vascular area (12). In patients with TIA, culprit plaques were identified if the symptoms corresponded to the affected vascular region. The location of the culprit plaque was determined by X. Yan and L. Li after being informed of clinical symptoms, TOF-MRA results, and DWI results, and independently evaluated. In cases of inconsistent assessments, another senior neuroradiologist, M. Tang, with 10 years of experience in imaging diagnosis, reassessed the images and assisted in reaching a consensus.

### 2.5 Statistical analysis

Data are reported as mean ± standard deviation or frequency (percentage). Student’s *t*-test, Mann–Whitney *U* test, χ^2^ test, or Fisher’s exact test was used to analyze differences in baseline clinical variables and plaque distribution burden between men and women. Given that the counting dependent variable was a discrete probability distribution, we used a Poisson regression model to analyze the relationship between sex and the burden of intracranial proximal, distal, and total plaques. The goodness-of-fit test was used to determine the over-discreteness of the counting dependent variable, and negative binomial regression was used for the regression model when the Pearson chi-square value divided by the degrees of freedom was >1. Least absolute shrinkage and selection operator (Lasso) regression was used for covariate selection in different models. Sex-related baseline clinical indicators (P < 0.1) or variables reported in the literature that might contribute to the effect were considered confounding variables and were included in LASSO regression for variable screening. The proportion of plaque distribution in male and female patients was interpreted as a risk ratio. Further, we constructed a similar regression model to analyze the interaction between sex and treatment in relation to plaque burden, i.e., to determine whether the plaque distribution count ratio between sexes differed depending on the treatment. The intra-class correlation coefficient was used to calculate the repeatability of the plaque measurement data. All statistical tests were two-sided, and a P-value <0.05 was considered statistically significant. Statistical analysis was performed using SPSS version 18 (SPSS Inc., Chicago, IL, USA) and the online platform SPSSPRO (http://www.spsspro.com).

## 3 Results

### 3.1 Demographic characteristics

Among the 278 patients initially assessed, 120 patients were finally included after excluding 19 patients with vasculitis, 17 patients with moyamoya disease, 11 patients with cardiogenic stroke; 39 patients with intracranial artery occlusion, 49 patients with thrombolysis, and 23 patients with obvious motion artifacts on HR-MRI images. (**Supplementary Figure S1**) The final cohort comprised 75 males and 45 females, with an average age of 60.77 ± 11.01 years. A total of 495 intracranial plaques were detected in 120 patients, including 311 proximal and 184 distal plaques. Male patients had higher levels of diastolic blood pressure (P = 0.011), and UA (P = 0.029) but lower levels of Apo A1 (P = 0.002), and HDL-C (P = 0.001) compared to female patients. In addition, the prevalence of current smoking was significantly higher in male patients than in female patients (P < 0.001) (**Table 1**). According to our findings, a high percentage of intracranial culprit plaques were located proximally in both male (85.3%) and female (88.9%) patients.

**Table 1:** Sex differences in terms of clinical features and atherosclerosis information in patients with type 2 diabetes with acute cerebral infarction.

Characteristics	All (N = 120)	Male (N = 75)	Female (N = 45)	*P*
Age (years)	60.77 ± 11.01	59.55 ± 11.35	62.80 ± 10.23	0.118
BMI (kg/m^2^)	24.59 ± 3.31	24.58 ± 3.30	24.63 ± 3.36	0.927
Clinical findings				
Systolic blood pressure (mmHg)	156.54 ± 29.56	158.01 ± 29.08	159.09 ± 30.51	0.484
Diastolic blood pressure (mmHg)	90.9(79.7,100.0)	90.0 (80.0, 100.0)	85.0 (74.5, 92.5)	0.011
Laboratory findings				
TC (mmol/L)	3.77 (3.15, 4.69)	3.64 (3.14, 4.65)	3.97 (3.18, 4.84)	0.295
TG (mmol/L)	1.38 (1.03, 1.80)	1.27 (0.99, 1.75)	1.05(1.15,1.96)	0.181
LDL-C (mmol/L)	2.2 (1.7, 2.8)	2.24 (1.64, 2.88)	2.19(1.76,2.77)	0.761
HDL-C (mmol/L)	1.00 (0.85, 1.17)	0.92 (0.81, 1.13)	1.07(0.92,1.35)	0.001
Apo A1	1.19 ± 0.22	1.14 ± 0.18	1.27 ± 0.24	0.004
Apo B	0.78 (0.64, 1.04)	0.79(0.66, 1.01)	0.78 (0.59, 1.06)	0.996
HbA1c %	7.67 ± 1.50	7.85 ± 1.68	7.34 ± 1.06	0.466
Fasting blood glucose	7.40 (5.89, 9.10)	7.23 (5.38, 10.02)	7.56(6.33,8.68)	0.501
Hcy (μmol/L)	13.9 (12.6, 17.2)	14.0 (12.8, 18.3)	13.6(10.6,16.3)	0.066
UA (mmol/L)	306.97 ± 88.74	320.71 ± 86.48	284.07 ± 88.67	0.029
Vascular risk factors N (%)				
Current smoker	31 (25.8%)	29 (38.6%)	2 (4.4%)	<0.001
History of HT	80 (66.7%)	49 (65.3%)	31 (68.8%)	0.689
Dyslipidemia	35 (29.2%)	19 (25.3%)	16 (35.6%)	0.233
Hyperuricemia ^a^	20 (16.7%)	10 (13.3%)	10 (22.2%)	0.206
History of stroke or TIA	38 (31.6%)	21 (28.0%)	17 (37.8%)	0.265
History of CHD/heart failure	26 (21.6%)	15 (20.0%)	11 (24.4%)	0.567
Hhcy ^b^	43 (35.8%)	29 (38.6%)	14 (31.3%)	0.403
Duration of diabetes (M)	30 (16,93)	30 (16,96)	30 (14,90)	0.784
Medications N (%)				
Statins	56 (46.7%)	37 (49.3%)	19 (42.2%)	0.450
Antiplatelet	55 (45.8%)	32 (42.7%)	23 (51.1%)	0.369
Antihypertensive	81 (67.5%)	50 (66.7%)	31 (68.9%)	0.803
Antidiabetic	66 (55.0%)	43 (57.3%)	23 (51.1%)	0.507
No. of plaques per vessel Segment N				
Total	495	343	152	–
Proximal	311	213	98	–
Distal	184	130	54	–
No. of culprit plaques per vessel segment N				
Proximal	104 (86.7%)	64 (85.3%)	40 (88.9%)	–
Distal	16 (13.3%)	11 (14.7%)	5 (11.1%)	–

Continuous variables are presented as means ± SDs or median (interquartile ranges).

BMI, body mass index; TC, total cholesterol; TG, triglyceride; LDL-C, low-density lipoprotein cholesterol; HDL-C, high-density lipoprotein cholesterol; Apo, Apolipoprotein; HT, hypertension; DM, diabetes mellitus; UA, uric acid; CHD, coronary heart disease; HHcy, hyperhomocysteinemia. ^b^HHcy ^>^15 μmol/L; ^a^Hyperuricemia: male >416 μmol/L, female >357 μmol/L.

### 3.2 Sex differences in intracranial plaque burden

**Figure 1:**
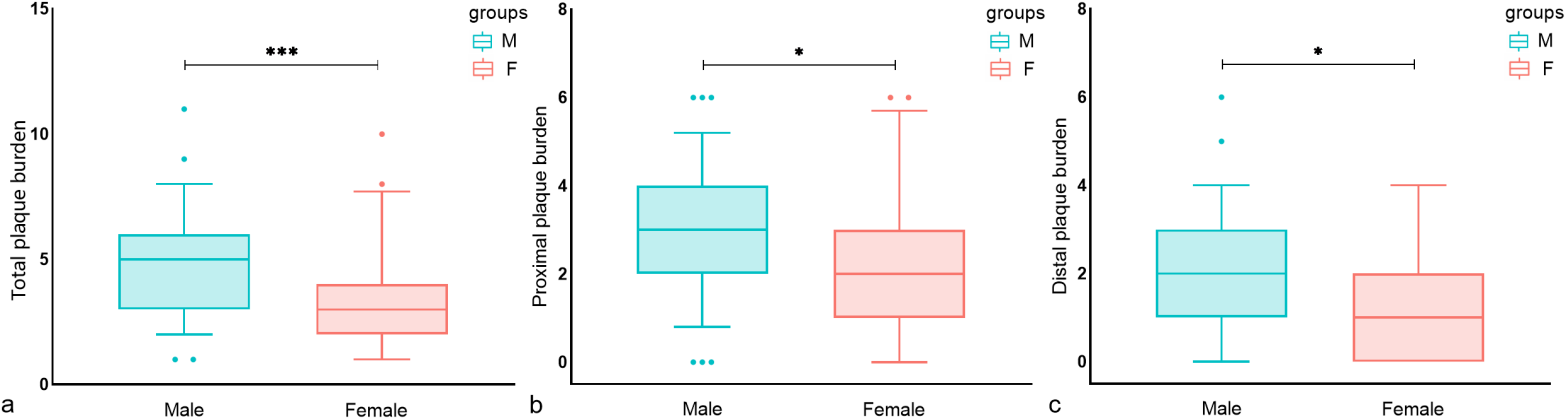
Sex differences in the distribution of intracranial plaque burden in T2DM patients. Box plots of **(A)** total intracranial plaque burden, **(B)** proximal plaque burden, and **(C)** distal plaque burden. significance was determined using a two-tailed Wilcoxon rank sum test. As shown in a–c, male patients had a higher total, proximal, and distal plaque burden compared to female patients. Circles represent data points beyond the whiskers. *P < 0.05, ***P < 0.001. M, Male; F, Female.

Univariate analysis showed that male patients had a higher total, proximal, and distal plaque burden compared to female patients (**Figure 1**). Multivariate regression analysis showed the adjusted risk of total and proximal intracranial plaque burden in male patients with diabetes mellitus with acute cerebral ischemia were 1.261 times (95%CI, 1.050–1.515, P = 0.013) and 1.332 times (95%CI, 1.055–1.682, P = 0.016) higher, respectively, compared to female patients with the condition. Although the difference was not statistically significant, the adjusted risk for distal intracranial plaque burden was also 1.316 times higher in males than in females (95%CI, 0.943–1.837, P = 0.106) (**Table 2**).

**Table 2:** Relationship between unadjusted and adjusted intracranial plaque burden and sex.

Outcome	Male	Female	Relative Risk P-value	Model
Total plaque burden	5.0 (3.0, 6.0)	3.0 (2.0, 4.0)	1.354 (1.115–1.644) 0.002	0
			1.261 (1.050–1.515) 0.013	1
Proximal plaque burden	3.0 (2.0, 4.0)	2.0 (1.0, 3.0)	1.304 (1.046–1.627) 0.019	0
			1.332 (1.055–1.682) 0.016	2
Distal plaque burden	2.0 (1.0, 3.0)	1.0 (0.0, 2.0)	1.444 (1.024–2.037) 0.036	0
			1.316 (0.943–1.837) 0.106	3

Model 0: unadjusted. Model 1: adjusted model with covariates age, diastolic blood pressure, uric acid levels, BMI, hcy, stains use, HbA1c%, duration of diabetes mellitus, and anti-diabetic treatment. Model 2: adjusted model with covariates age, diastolic blood pressure, current smoking status, uric acid levels, duration of diabetes mellitus, anti-diabetic treatment, and HbA1c%. Model 3: adjusted model with covariates age, BMI, duration of diabetes mellitus, hcy, diastolic blood pressure, HbA1c%, antidiabetic treatment, and uric acid levels.

### 3.3 Sex-specific effects of antidiabetic treatment on intracranial plaque burden

**Figure 2:**
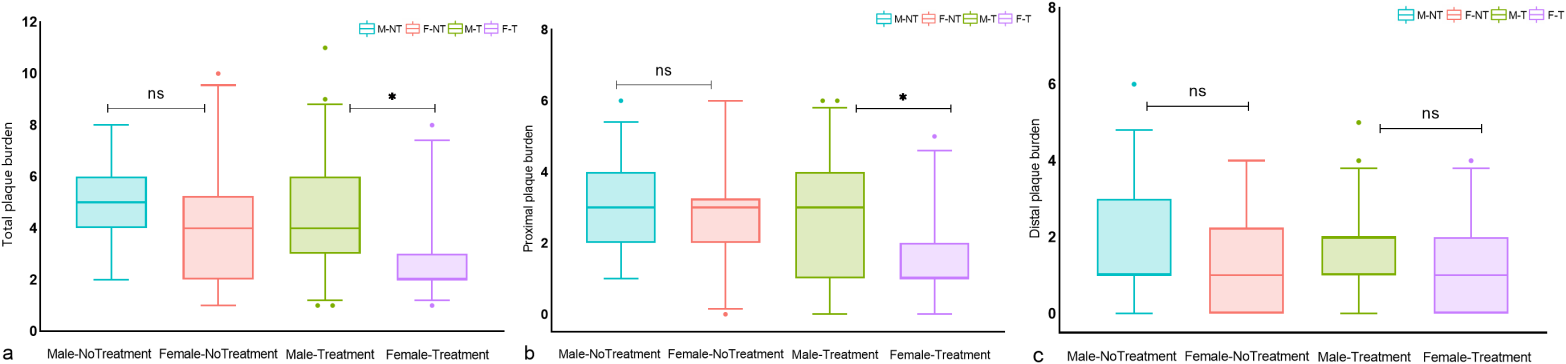
Differences in intracranial plaque burden between treated and untreated male and female T2DM patients. Box plots of **(A)** total intracranial plaque burden, **(B)** proximal plaque burden, and **(C)** distal plaque burden; significance was determined using a two-tailed Wilcoxon rank sum test. As shown in **(A–C)**, there was no significant difference in total, proximal, and distal plaque burden between untreated men and women. In the treatment group, a significant sex difference was observed in total and proximal intracranial plaque burden, but not in distal plaque burden. Circles represent data points beyond the whiskers. *P < 0.05, ns P > 0.05. M-NT, Male-No Treatment; F-NT, Female-No Treatment; M-T, Male-Treatment; F-T, Female-Treatment. ns means not significant P>0.05.

There were no significant differences between untreated males and female patients in terms of total, proximal, and distal plaque burden. In the treatment group, a significant sex difference was observed in total and proximal intracranial plaque burden, but not in distal plaque burden. (**Figure 2**) In the multivariate regression analysis of the impact of sex on antidiabetic treatment outcomes, as shown in **Table 3**, female patients undergoing treatment for diabetes showed a significant reduction in intracranial proximal plaque burden, with a risk ratio of 1.530 (95%CI, 1.076–2.174) when comparing male to female patients. In contrast, in the untreated setting, females were at risk for proximal plaque burden, with a male-to-female risk ratio of 0.966 (95% CI: 0.704-1.324). The interaction test examining the combined effect of sex and treatment found that the relative difference between the two risk ratios was 1.579 (95%CI, 1.063–2.346, P = 0.024). A similar trend was found in the total plaque burden, although there was no significant difference in the interaction analysis.

**Table 3:** Impact of sex on intracranial plaque burden and antidiabetic treatment.

Subgroups	Male	Female	Relative risk	Treatment/no treatment relative risk ratio	Model
Total, treatment	4.0 (3.0, 6.0)	2.0 (2.0, 3.0)	1.437 (1.063–1.776)	1.376 (0.987–1.917) P = 0.059	1
Total, no treatment	5.0 (4.0, 6.0)	4.0 (2.0, 5.25)	1.136 (0.856–1.509)		
Proximal, treatment	3.0 (1.0, 4.0)	1.0 (1.0, 2.0)	1.530 (1.076–2.174)	1.579 (1.063–2.346) P = 0.024	2
Proximal, no treatment	3.0 (1.0, 3.0)	3.0 (2.0, 3.25)	0.966 (0.704–1.324)		
Distal, treatment	2.0 (1.0, 2.0)	1.0 (0.0, 2.0)	1.216 (0.836–1.769)	1.095 (0.568–2.186) P = 0.787	3
Distal, no treatment	1.5 (1.0, 3.0)	1.0 (0.0, 2.25)	1.424 (0.826–2.455)		

Model 1: adjusted model with covariates age, BMI, diastolic blood pressure, uric acid levels, hcy, stains use, HbA1c%, and duration of diabetes mellitus. Model 2: adjusted model with covariates age, diastolic blood pressure, current smoking status, uric acid levels, duration of diabetes mellitus, and HbA1c%. Model 3: adjusted model with covariates age, BMI, duration of diabetes mellitus, diastolic blood pressure, hcy, HbA1c%, and uric acid levels.

### 3.4 Reproducibility assessment

**Table 4** summarizes the inter-observer reproducibility data. All measurements showed excellent inter-observer agreement.

**Table 4:** Inter-observer reproducibility.

Parameters	Inter-observer	
	Intra-class correlation coefficients	95%CI
Male		
Total	0.949	0.920-0.967
Proximal	0.938	0.903-0.960
Distal	0.962	0.941-0.976
Female		
Total	0.914	0.849-0.952
Proximal	0.951	0.913-0.973
Distal	0.935	0.884-0.964

## 4 Discussion

The main findings of our study are as follows (1): In patients with T2DM with acute cerebral ischemia, the adjusted risk of total and proximal intracranial plaque burden was significantly higher in males than in females (2). Treatment for diabetes has a sex-specific effect on the burden of intracranial proximal plaques. Females treated with glucose-lowering drugs experienced a more significant reduction in proximal intracranial plaque burden compared to males. Notably, we found that more than 85% of the intracranial culprit plaques in both male and female patients were located proximally in this study. This was similar to the findings of Wu et al. In a case-control study using VW-MRI to identify intracranial plaques, data showed that most of the culprit plaques in the first-time and recurrent stroke groups were located in the proximal segment of the artery (13). Similar results were observed in the distribution of culprit plaques in coronary and internal carotid arteries. Internal carotid artery bulbar (proximal) plaque is associated with the risk of major adverse cardiovascular events (14). Culprit plaques that cause acute myocardial infarction tend to accumulate at the proximal segment of the coronary branches (left anterior descending and left circumflex branches) (15, 16). It follows that understanding how to reduce the plaque burden in these areas may help reduce the risk of cerebrovascular disease. Further prospective studies are needed to confirm these preliminary results and provide evidence for improved management of gender-related clinical differences in T2DM.

Data from Western European and Asian populations suggest that the prevalence of T2DM is higher in men than in women (17). The prevalence of T2DM increases with age in both sexes (18). In individuals aged >63 years, the prevalence of T2DM as well as the incidence of atherosclerotic disease rises sharply, especially in women (19). This trend may be partly attributed to the influence of estrogen (20). T2DM has been reported to be associated with a higher number of vessel wall lesions in the anterior circulation (RR = 1.67; 95%CI, 1.20–2.33) (21) and an increased number of intracranial artery stenoses (OR = 2.4; 95%CI, 1.04–5.57, P = 0.04) (22), suggesting that the burden of atherosclerosis is strongly associated with the prevalence of T2DM. We found that the sex difference in intracranial atherosclerosis burden had a similar trend to that in the prevalence of T2DM. In our study population with a mean age of 61.15 years, both overall and proximal plaque burdens were significantly higher in men with T2DM compared to women. On the one hand, this disparity may be related to the cardiovascular protective effects of estrogen in early postmenopausal women (23). Autopsy studies have found that the prevalence of ICAS increased in women after the age of 65 years, gradually reaching levels comparable to those observed in men (24). Estrogen can delay the onset of atherosclerotic plaques (25). Our results are similar to those of a recent 7T-MRI-based *in vivo* study, wherein Shozushima et al. reported that the coexistence of plaque in anterior and posterior intracranial circulation was associated with male sex in patients with type 2 diabetes mellitus, with a mean age of 53.2 ± 6.3 years (26). Even in the extracranial carotid artery, males with T2DM have a higher severity of atherosclerosis, as measured by carotid intimal thickness and plaque score, compared to females (27, 28). On the other hand, in subgroup univariate analysis, we found no significant differences in total, proximal, and distal intracranial plaque burdens between male and female patients not treated for diabetes mellitus, whereas in the treatment group, the total and proximal intracranial plaque burdens were higher in male patients than in female patients, which resembles the sex differences in intracranial plaque burdens in the overall study population. Although the cardiovascular protective effects in women may extend to those with type 2 diabetes (1), the hormonal anti-inflammatory and neuroprotective effects of hormones in older women diminish with the gradual decrease in estrogen levels in postmenopausal women, coupled with the high-inflammatory environment produced by diabetes (25). Therefore, in this study population, anti-diabetes treatment has a potential impact on sex differences in intracranial plaque burden.

The high burden of ICAS may be an independent biomarker reflecting long-term vascular risk (8). Although sex differences in the incidence and mortality of adverse cardiovascular events in patients with T2DM have been identified, there have been few studies on sex-specific impacts of hypoglycemic drug interventions, particularly on direct outcomes or surrogate endpoints such as cerebral atherosclerotic burden. Our study provides important evidence in this regard. We found a significant gender-specific effect of glucose-lowering therapy on the reduction of proximal plaque burden in intracranial arteries. Considering that most of the culprit plaques causing ischemic symptoms are located proximally, further prospective studies are needed to determine whether women have a more favorable cerebrovascular risk reduction after treatment compared to men. Such findings would be instrumental in guiding gender-tailored treatment strategies for individuals with T2DM. In particular, previous studies have found that glycemic control is more challenging in women, especially older women, than in men (25).

Studies have shown that estrogen mediates endothelial function and improves atherosclerosis by acting on estrogen receptors, and the expression of estrogen receptors in vascular smooth muscle and endothelial cells is higher in women than in men (29). In addition, hyperglycemia-induced overproduction of reactive oxygen species (ROS) triggers persistent epigenetic changes in proinflammatory factors. This phenomenon, known as “hyperglycemic memory,” drives the persistent expression of these factors even after the normalization of blood glucose levels. It is suggested that this metabolic memory mediates the inflammatory responses that cause persistent endothelial cell damage even when blood glucose levels are lowered after treatment, thereby influencing the progression of atherosclerotic lesions (30, 31). In women with diabetes mellitus, effective control of hyperglycemia may activate the estrogen receptor α pathway, which is known to mediate the antiproliferative effects on vascular endothelial cells (32), protect endothelial cells from ROS, inhibit their activity, and impede their pro-inflammatory effects, thereby attenuating the atherosclerotic process (33). Therefore, differences in estrogen receptor expression may be able to explain the observed sex-specific associations between atherosclerotic burden and antidiabetic therapy in our study.

In addition, we observed that in the treatment group, women (HbA1c < 7%) showed better glycemic control compared to men (HbA1c > 7%) (**Supplementary Table S1**). Strang et al. found that after two years of cardiovascular disease prophylaxis, targeting glycosylated hemoglobin levels below 7% (53 mmol/mol), atherosclerosis resolved in patients with type 2 diabetes mellitus (9.6% reduction in wall volume, P = 0.016) (34). Similarly, Huang et al. found that compared to patients with diabetes with controlled glycemia, those with uncontrolled glycemia (HbA1c level ≥ 7.0%) had significantly greater maximum plaque length (P < 0.05) (3). These results suggest that the degree of glycemic control affects the severity of intracranial atherosclerotic disease (35). Shah et al. found that poorer glycemic control was independently associated with carotid atherosclerosis progression in young patients with type 2 diabetes, and that this adverse effect was more pronounced in men (36). In addition, although not statistically significant, we found that the percentage of regular treatment was higher in females than in males in our study, which may suggest that treatment adherence is higher in females than in males. A retrospective cohort study in Japan that included 884 patients with T2DM found that male sex was an independent risk factor for poor adherence to oral hypoglycemic medications (37). Conversely, another study reported higher medication adherence among men with T2DM compared to women (38). Although the relationship between sex and medication adherence remains to be determined, variations in glycemic control as well as treatment adherence in this study may provide additional explanations for the observed sex differences in the burden of intracranial atherosclerosis. Notably, sex differences in drug management and treatment response for T2DM suggest that undertreatment is a major issue for women. This explains why women with T2DM have a relatively poor prognosis for macrovascular complications such as stroke (25). In our study, the interaction analysis examining the burden of ICAS in relation to sex and treatment in patients with T2DM with acute cerebrovascular disease showed that women may be more likely to benefit from aggressive diabetes treatment strategies.

This study also had some noteworthy limitations. First, there was an imbalance in the number of male and female participants, which may be related to several reasons, such as differences in the prevalence of T2DM as well as cerebrovascular disease, bias in receiving timely imaging evaluations, or variations in willingness to participate in the study. Second, the retrospective cross-sectional nature of the study with a relatively small sample size restricts our ability to draw causal inferences from the observed results. Therefore, future studies should adopt a prospective longitudinal experimental design with a more balanced representation of male and female participants; include detailed information on sex hormones and menopausal status in patients with T2DM; and analyze the sex differences in macrovascular complications, plaque burden, and treatment outcomes in a more representative patient cohort. Third, the duration of diabetes in this study was based on the timing of the first diagnostic test. Since patients may rarely go to the hospital for blood glucose testing until symptom onset, the actual duration of diabetes could be underestimated. In addition, we did not collect patient information about pre-diabetes, which may also influence the assessment of atherosclerosis burden. Finally, the patients included in the study were from an Asian population; hence, the results need to be interpreted with caution in other ethnic groups.

## 5 Conclusion

Male patients with T2DM who experienced acute cerebrovascular disease had a significantly higher adjusted risk of intracranial total and proximal atherosclerotic burden compared to female patients. Furthermore, Female patients undergoing antidiabetic treatment have a significantly reduced risk of proximal plaque compared to males.

## Data Availability

The original contributions presented in the study are included in the article/**Supplementary Material**. Further inquiries can be directed to the corresponding author. Requests to access the datasets should be directed to DZ, yunyun122424@126.com.
