# Supplementary material for: Sex differences in intracranial plaque burden in patients with type 2 diabetes mellitus with acute ischemic cerebrovascular disease: a pilot study based on high-resolution MRI
Source: Front Endocrinol (Lausanne). 2025 Jan 24;15:1417240. doi: 10.3389/fendo.2024.1417240 (PMC11802420; doi:10.3389/fendo.2024.1417240)
Supplement: Supplementary file 1 [file DataSheet1.docx]

Supplementary Material


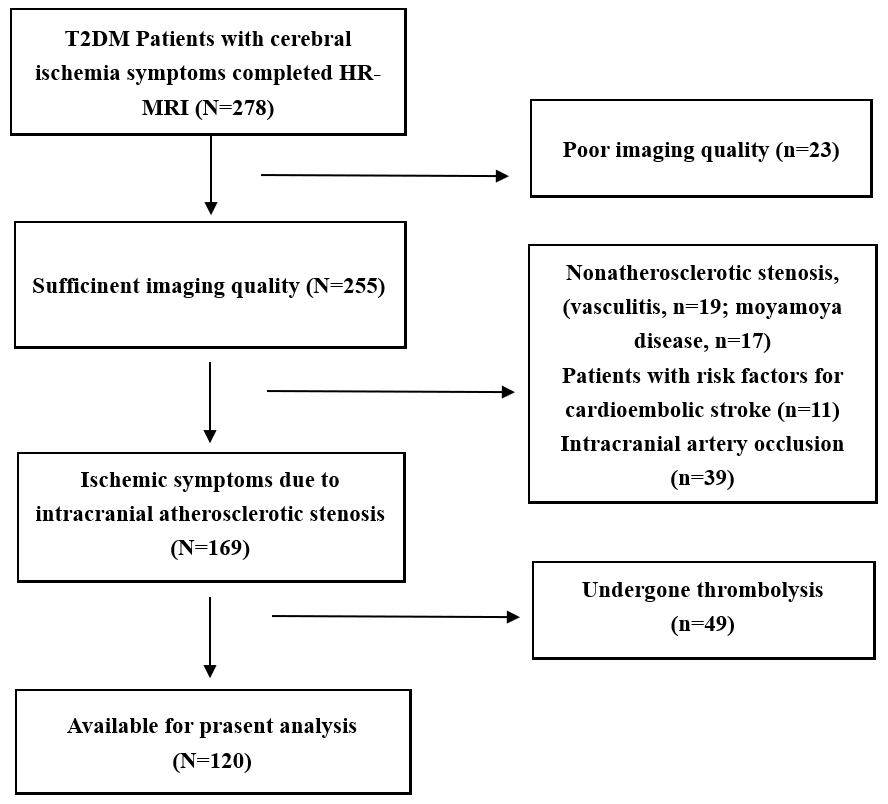


**Supplementary Figure 1:** Flow chart of patient recruitment for final analysis.
