# Supplementary material for: Sex differences in intracranial plaque burden in patients with type 2 diabetes mellitus with acute ischemic cerebrovascular disease: a pilot study based on high-resolution MRI
Source: Front Endocrinol (Lausanne). 2025 Jan 24;15:1417240. doi: 10.3389/fendo.2024.1417240 (PMC11802420; doi:10.3389/fendo.2024.1417240)
Supplement: Supplementary file 2 [file Table1.docx]

Supplementary Material

Table S1. Differences in diabetes-related indicators between treated and untreated male and female patients

|  | No-treatment | |  | Treatment | |  |
| --- | --- | --- | --- | --- | --- | --- |
| Characteristic | Male | Female | P | Male | Female | P |
|  | N = 32 | N = 22 |  | N = 43 | N = 23 |  |
| Age (years) | 57.38 ± 12.56 | 61.73 ± 13.02 | 0.223 | 61.42 ± 10.38 | 64.91 ± 7.63 | 0.212 |
| BMI (kg/m^2^) | 25.87 ± 3.93 | 26.00 ± 3.41 | 0.898 | 23.62 ± 2.37 | 23.33 ± 2.80 | 0.659 |
| HbA1c (%) | 8.30 ± 1.86 | 7.82 ± 1.22 | 0.765 | 7.53 ± 1.47 | 6.89 ± 0.63 | 0.374 |
| Fasting blood glucose | 7.8(5.4, 10.5) | 7.6(6.7, 9.2) | 0.958 | 7.21 ± 2.40 | 7.52 ± 2.08 | 0.476 |
| Duration of diabetes (months) | 18 (15, 24) | 20.5 (10.3,30.5) | 0.620 | 84 (24, 120) | 84 (36, 120) | 0.570 |
| Antidiabetic medication N% | -- | -- |  |  |  |  |
| Metformin |  |  |  | 26 (60.5%) | 13 (56.5%) | 0.756 |
| Sulphonylurea |  |  |  | 16 (37.2%) | 8 (34.8%) | 0.845 |
| Thiazolidinedione |  |  |  | 5 11.6%) | 5 (21.7%) | 0.275 |
| Insulin |  |  |  | 8 (18.6%) | 4 (17.4%) | 0.903 |
| GLP-1RA |  |  |  | 13 (30.2%) | 6 (26.1%) | 0.723 |
| SGLT-2 |  |  |  | 8 (18.6%) | 2 (8.6%) | 0.285 |
| Regular antidiabetic treatment N% | -- | -- |  | 30 (69.8%) | 20 (86.9%) | 0.121 |

*Continuous variables are presented as means±SDs or median (interquartile ranges).*

*GLP-1RA = glucagon-like peptide 1 receptor agonist; SGLT-2 = sodium-glucose co-transporter 2 inhibitors.*
